# Epigenetic Aspects of Engineered Nanomaterials: Is the Collateral Damage Inevitable?

**DOI:** 10.3389/fbioe.2019.00228

**Published:** 2019-09-20

**Authors:** Mallikarjuna Rao Gedda, Piyoosh Kumar Babele, Kulsoom Zahra, Prasoon Madhukar

**Affiliations:** ^1^Department of Biochemistry, Institute of Science, Banaras Hindu University, Varanasi, India; ^2^Department of Chemical and Biomolecular Engineering, Vanderbilt University, Nashville, TN, United States; ^3^Department of Biochemistry, Institute of Medical Sciences, Banaras Hindu University, Varanasi, India; ^4^Department of Zoology, Institute of Science, Banaras Hindu University, Varanasi, India

**Keywords:** nanomaterials (ENMs), nanotoxicity, epigenetic modifications, nanotheranostics, protein-corona, nano-risk assessment

## Abstract

The extensive application of engineered nanomaterial (ENM) in various fields increases the possibilities of human exposure, thus imposing a huge risk of nanotoxicity. Hence, there is an urgent need for a detailed risk assessment of these ENMs in response to their toxicological profiling, predominantly in biomedical and biosensor settings. Numerous “toxico-omics” studies have been conducted on ENMs, however, a specific “risk assessment paradigm” dealing with the epigenetic modulations in humans owing to the exposure of these modern-day toxicants has not been defined yet. This review aims to address the critical aspects that are currently preventing the formation of a suitable risk assessment approach for/against ENM exposure and pointing out those researches, which may help to develop and implement effective guidance for nano-risk assessment. Literature relating to physicochemical characterization and toxicological behavior of ENMs were analyzed, and exposure assessment strategies were explored in order to extrapolate opportunities, challenges, and criticisms in the establishment of a baseline for the risk assessment paradigm of ENMs exposure. Various challenges, such as uncertainty in the relation of the physicochemical properties and ENM toxicity, the complexity of the dose-response relationships resulting in difficulty in its extrapolation and measurement of ENM exposure levels emerged as issues in the establishment of a traditional risk assessment. Such an appropriate risk assessment approach will provide adequate estimates of ENM exposure risks and will serve as a guideline for appropriate risk communication and management strategies aiming for the protection and the safety of humans.

## Introduction

The environment surrounding us has a plethora of natural and manmade toxicants. These toxicants have gained access to almost every aspect of our life such as air, water, food, homes, workplaces, and belongings. It is very difficult to detoxify and eliminate toxicants after their entry into the human body, leading to the development of different syndromic symptoms. The exposure to these toxicants causes severe illness, diseases, and disability or death. Some of the common environmental toxicants are chemical compounds such as pesticides, heavy metals, plastics, and hydrocarbons. Many of these chemical compounds possess carcinogenic properties and, due to their common use in our society, are unavoidable. The jumbled chaos and rapid industrialization have profound effects on the augmentation of the air, water, and soil pollution. One of the rapidly emerging research areas in the twenty-first century is nanotechnology. Due to the advent and advancement of the nanoscience and technologies many engineered nanomaterials (ENMs) have been given access to our lives. ENMs have a broad spectrum of applications in various fields, such as in the fields of nanotheranostics and personalized medicine (the field of delivering a suitable drug to the right subjects at a precise time). The use of personalized medicine for cancer therapy is one of the promising areas in nanotheranostics (Yaari et al., 2016). Due to their widespread application, manufacture, and disposal, ENMs are released into the natural environment and so their accidental consumption is inevitable. The appearance of ENMs in the soil, water, and air could pose harmful threats for both humans and the environment, leading to serious health issues (Singh and Singh, 2019). Over recent years, it has been observed that epigenetic modifications greatly influence human physiology and development. There are innumerable pieces of evidence of epigenetic dysregulation in several human diseases, especially cancer, and much of drug discovery research is focusing on epigenetics (Strahl and Allis, 2000; Robertson, 2005; Portela and Esteller, 2010; Chervona and Costa, 2012). Therefore, the concept of personalized nanomedicine is modestly improved by epigenetic biomarkers. A large number of ENMs are being used as drug carriers in personalized medicine for drug delivery and diagnosis. These drugs significantly improve drug delivery to targeted cells as compared to the free diffusion of drug molecules. Although these drug delivery systems are advantageous over conventional chemotherapy, the substantial unidentified issues and potential cytotoxicity associated with the ENMs cannot be ignored (Oberdörster et al., 2005). The nanoscale materials have diverse properties and hence distant toxicity parameters in comparison to their larger counterparts. The destruction of cancerous cells by ENMs-based drug delivery system selectively removes the tumor. However, ENM associated non-cancerous cell dysfunctions and their repercussions can't be ignored, therefore nanomedicine and nanotoxicity have a strong correlation. The understanding of the interconnections between ENMs-based drug administration and the related toxicity greatly broadens our understanding of therapeutic strategies because ENMs share similar biological fates/responses in the body.

**Figure 1:**
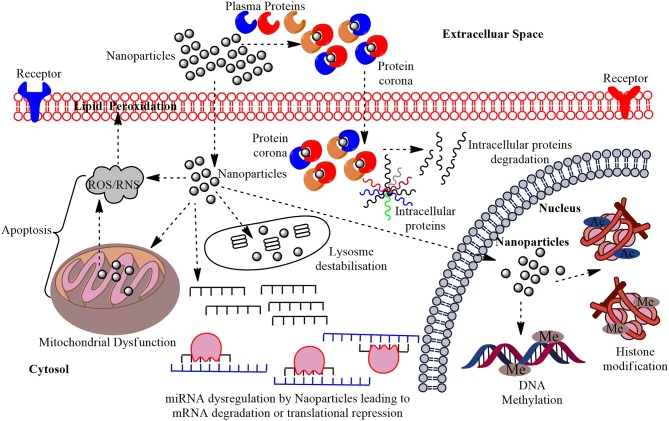
The multi-layered epigenetic effects of Nanomaterials: Engineered metallic and non-metallic nanomaterials gain entry into the cells and may directly interact with the genetic material or may affect intermediate molecules indirectly leading to cytotoxicity, genotoxicity, peroxidation of lipids, apoptosis, and dysregulation of miRNAs; altered expression of their associated genes. NPs gain entry into the PM of the target cells in conjugation with the plasma proteins and forms protein corona and this can induce cytotoxicity by making a path for the degradation of intracellular proteins. Interaction of NPs with the mitochondria leads to the production of Reactive Oxygen Species (ROS) or Reactive Nitrogen Species (RNS) causing oxidative stress and affects the PM through lipid peroxidation which eventually leads to apoptosis. The miRNA gets dysregulated on interaction with NPs leading to altered expression of their associated genes and functional proteins. The major epigenetic modification by NPs include histone modification and DNA methylation, which may cause epigenetic effects in the cell.

The toxicity caused by ENMs affects organisms ranging from prokaryotes to higher eukaryotes, including humans, and is well-documented (Singh and Singh, 2019). The exposure of ENMs has been well-studied in various *in vivo* and *in vitro* assays leading to cytotoxicity, genotoxicity, peroxidation of lipids, micronuclei formation, apoptosis, and altered expression of associated genes as shown in Figure 1. The other adverse effects of ENMs are the inflammatory response, reproductive toxicity, immunotoxicity, and non-genotoxic carcinogenicity (Dusinska et al., 2017). Recent studies utilizing modern “-omics” (proteomics and metabolomics) technologies were successful in drawing significant conclusions of underlying mechanisms of nanotoxicity (Babele, 2019; Babele et al., 2019). The majority of the nanotoxicity studies are conducted at genetic levels and some of the proposed mechanisms for toxicity are altered gene and protein expressions of major cellular pathways (Babele et al., 2018). However, epigenetic variations have gained relatively little attention. The mechanism of modulation of epigenetic processes dependent on cellular and potentially complex disease is a promising topic. The understanding of the interaction occurring at the interface between ENMs and biological components is necessary to predict the fate and concern of these injected ENMs and addressing the concerns of ENM based drug targeting. To illustrate a classical mechanism that maintains the epigenetic state, a little information about epigenetic modification is must and our review provides a detailed account of the fundamental principles and concepts.

## Mechanism of ENMs Internalization

**Figure 2:**
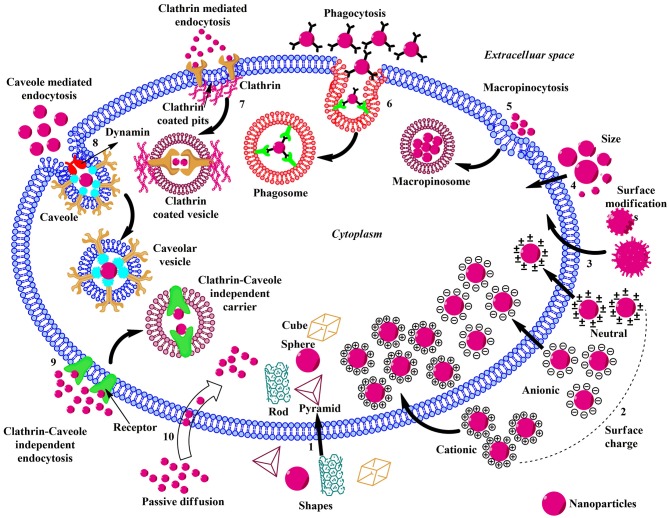
Schematic illustration of the influence of physicochemical properties, endocytic, and non-endocytic pathways on the cellular uptake of ENMs. (1) The shape of the ENMs play pivotal role in the uptake and there are considerable amounts of variations in the translocation rate of rod, cube, pyramid, and sphere-shaped ENMs. (2) Charged ENMs show better uptake than the uncharged. The positively charged ENMs internalize via macropinocytosis and negatively charged NPs enter via clathrin/caveolae independent endocytosis. (3) ENMs in the size range of 30–50 nm interact efficiently with the receptors on the plasma membrane and show rapid uptake via receptor-mediated endocytosis. (4) Surface modification of ENMs involve coating their surface with PEG, cationic (–NH_2_), anionic (–COOH), or neutral (–OH) molecules to enhance uptake and reduce toxicity. (5) Macropinocytosis; upon recognition, the ENMs and surrounding extracellular fluid are entrapped in large vesicles (macropinosome) formed by the back fusion of large membranous extensions. (6) Phagocytosis; Antibodies or complement proteins (opsonins) get adsorbed on the surface of ENM and the opsonized particles are recognized through receptors present on phagocytic cells and get internalized. (7) Clathrin-mediated Endocytosis; The ENM binds with the surface receptor present within the clathrin-coated pits inducing invagination and the vesicle is released into the cytoplasm by the help of scission protein; dynamin. (8) Caveolae-mediated Endocytosis; Caveolin proteins form a flask-like curvature with the trapped ENM and the resulting vesicle is released into the cytoplasm by dynamin. (9) Clathrin/Caveole independent Endocytosis- takes place in the cells lacking clathrin or caveolae and it is a non-destructive uptake mechanism which bypasses the lysosomal hydrolysis. (10) Passive Diffusion- is a non-endocytic uptake mechanism, mainly utilized by the DPA-QDs.

Understanding of the underlying mechanism(s) involved in cellular uptake and intracellular trafficking of ENMs is essential for evaluating the biomedical function, bio-distribution, toxicity, and therapeutic efficacy (Behzadi et al., 2017; Foroozandeh and Aziz, 2018). The various factors that affect the uptake of ENMs have been extensively covered in the following section and illustrated in Figure 2. The insight into the role of physicochemical parameters such as size, shape, charge, hydrophobicity/hydrophilicity, and surface functionalization on internalization is essential as these properties directly alter the uptake level, mode of endocytosis, and cytotoxicity of ENMs. The membrane permeability and integrity rely largely on the size and surface chemistry of interacting ENMs, and a particular type may utilize multiple endocytic pathways depending on its size. ENMs with sizes ranging from few to several hundred nanometers internalize via pinocytosis/macropinocytosis, and those with sizes ranging from 250 nm to 3 μm undergo phagocytosis, while ENMs in the size range of 120–150 nm and even 200 nm internalize through clathrin- or caveolin-mediated endocytosis. Larger ENMs internalize with great difficulty via caveolae-mediated pathway due to hindrance caused by the size of caveolae (Lu et al., 2009). The optimum size at which a particular ENM shows most efficient internalization with a higher uptake rate is 50 nm, while the uptake is reduced with sizes larger or smaller than this; although the clearance rate of larger ENMs is much faster than that of smaller ones (Osaki et al., 2004; Geiser et al., 2005; Chithrani and Chan, 2007; Jin et al., 2009; Lu et al., 2009; Wang et al., 2010).

The shape of ENMs is another crucial factor in its cellular uptake. Several experiments have shown that rod-shaped ENMs undergo lower cellular uptake than spherical ENMs (Chithrani et al., 2006; Chithrani and Chan, 2007). On the contrary, Gratton et al., while working on monodisperse hydrogel particles, demonstrated that rod-shaped ENMs have the highest internalization rates in HeLa cells compared to spheres, cylinders and cubes (Gratton et al., 2008). In a recent study on rod-shaped polystyrene NPs on Caco-2 cells, two-fold greater uptake of rod and disc-shaped NPs was reported (Banerjee et al., 2016). A study by Herd et al. revealed that spherical NPs undergo clathrin-mediated endocytosis while their worm-like counterparts internalize via micropinocytosis or phagocytosis due to their large sizes (Herd et al., 2013).

The charge on the surface of ENM is a crucial factor influencing its cellular uptake and recently, several attempts are being made to engineer the surface charge of NP rendering it cationic or anionic (Zhu et al., 2012). The negative charge on the plasma membrane enhances the uptake of positively charged ENMs. Many studies demonstrated that positively charged ENMs have higher uptake rates and are internalized via macropinocytosis than negatively charged ENMs which prefer clathrin-/caveolae-independent endocytosis (Dausend et al., 2008; Marano et al., 2011; Panariti et al., 2012). The uptake of positively charged ENM is believed to increase the fluidity of the cell membrane while negatively charged ENM causes gelation of the membrane and has higher uptake rate than the neutral ones (Wang et al., 2008; Arvizo et al., 2010). Charged ENMs show better adhesion to the membrane bilayer than the uncharged.

The ENMs with hydrophobicity show highest thermodynamic stability in the middle of the lipid bilayer while the hydrophilic particles get lodged on the surface, which has been observed in both AuNPs (Lee et al., 2013) and quantum dots (Olubummo et al., 2012). Surface modification and elasticity also play a critical role in cellular uptake. Stiffer ENMs internalize more rapidly and efficiently as compared to the softer ones (Anselmo et al., 2015). Gold, QDs, and magnetic ENMs are hard with higher elastic values, while biodegradable polymers, liposomes, and hydrogels are soft ENMs with lower elastic values. Surface modifications by functionalizing ENMs with PEG (polyethylene glycol), negatively charged groups like carboxyl (–COOH), positively charged amino (–NH_2_) group and neutral hydroxyl (–OH) groups help in reducing toxicity, enhancing stability and improving cellular internalization (Chompoosor et al., 2010). The enhancement of positive surface charge enhances the cellular uptake of ENMs (Holzapfel et al., 2006; Alexis et al., 2008). Likewise, COOH functional groups increase the negative charge of ENMs and boost its uptake (Holzapfel et al., 2006).

### Cellular Uptake Pathways of ENMs

ENMs are mostly small, polar molecules that utilize the endocytic pathway to infiltrate into the cells, which can be categorized into phagocytosis and pinocytosis. The pinocytic mode can be sub-categorized into caveolae-mediated, clathrin-mediated, clathrin- and caveolae-independent endocytosis and macropinocytosis (Behzadi et al., 2017; Foroozandeh and Aziz, 2018). All types of endocytic and non-endocytic pathways for ENMs internalization are summarized below.

#### Phagocytosis

Larger ENMs such as polystyrene, radiolabeled albumin, polymer–lipid hybrid nanoparticle (PLN), PEGylated gold nanorods, and nanospheres, ranging in a size of 200–2,100 nm are efficiently uptaken by phagocytes by adsorption of opsonins such as immunoglobulin, laminin, fibronectin, or complement proteins to the surface of ENMs (Caviston and Holzbaur, 2006; Hillaireau and Couvreur, 2009; Xiang et al., 2012; Pauwels et al., 2017). Opsonized ENMs are easily recognized by phagocytes via specific ligand-receptor interaction. The receptors present on the surface of phagocytes are complement receptors, Fc receptors, mannose/fructose receptors, and scavenger receptors. The opsonized ENM–phagocyte complex initiates a signaling cascade leading to actin polymerization, formation of cell surface extensions, engulfing, internalization, and formation of phagosome. ENMs with different charges attract opsonins leading to enhanced phagocytosis in comparison with the uncharged ones.

#### Clathrin-Mediated Endocytosis

Poly (lactic-co-glycolic acid), D, L-polylactide, poly(ethylene glycol-co-lactide), Silica-based (SiO_2_), Herceptin-coated gold, Carbon Nanotubes (CNTs), Silica Nanotubes (SNTs), polystyrene, carboxylated polystyrene, alginate–chitosan, carboxylated quantum dots, and almost all lipid-based ENMs internalize via Clathrin-mediated endocytosis (Behzadi et al., 2017; Foroozandeh and Aziz, 2018). This process involves the binding of ligand in the extracellular fluid to the low-density lipoprotein receptor on the cell membrane forming ligand-receptor complex which migrates to that region of the cell membrane, which is rich in clathrin (0.5–2% of the cell surface) and gets engulfed through the formation of clathrin-coated vesicles (Conner and Schmid, 2003; Doherty and McMahon, 2009; Capraro et al., 2013; Vanlandingham et al., 2014; Lu R. et al., 2016; Ferguson et al., 2017; Hassinger et al., 2017). On internalization, the clathrin coat is removed followed by fusion of the cargo with endosomes and finally with lysosomes.

#### Caveolae-Mediated Endocytosis

Amine labeled polystyrene (60 nm), alginate-chitosan (157 nm), silica 60 nm), and polystyrene NP (40 nm) utilize this mode of internalization (Behzadi et al., 2017; Foroozandeh and Aziz, 2018). Caveolae (flask-shaped membranous invaginations ranging from 50 to 80 nm) composed of membrane protein caveolin-1 are distributed in the regions of dense bodies anchoring the cytoskeleton in epithelial cells, endothelial cells, fibroblast cells, adipocytes, and smooth muscle cells (Thorn et al., 2003; Parton and Simons, 2007; Wang et al., 2011). After detaching from the plasma membrane, caveolae fuse with cellular compartment called caveosomes, which exist at neutral pH and are able to bypass the lysosome preventing the hydrolytic degradation and thus useful for the internalization of ENMs.

#### Clathrin/Caveolae Independent Endocytosis

Clathrin/caveolae independent endocytosis is mainly shown by folate functionalized ENMs (Foroozandeh and Aziz, 2018). The binding of folate-modified ENM to its receptor leads to its non-destructive delivery into the cytoplasm. These cargos often remain in endocytic compartments bypassing the lysosome. The clathrin/caveolae independent endocytosis occurs in cells lacking clathrin and caveolae and is mostly used by growth hormone, Interleukin-2 and glycosylphosphatidylinositol linked proteins to internalize (Damm et al., 2005; Kirkham et al., 2005; Soldati and Schliwa, 2006; Mellman and Nelson, 2008; Sandvig et al., 2011; Ferreira and Boucrot, 2018; Zhang F. et al., 2018).

#### Macropinocytosis-

Micron-sized ENMs like polystyrene (40 nm), carboxylated polystyrene (40 and 200 nm) and lipid ENMs (60 nm) are translocated through this mode (Behzadi et al., 2017). The rearrangement of the cytoskeleton led to the formation of large membranous extensions or ruffles which forms large vesicles on fusion with the cell membrane, trapping a large amount of extracellular fluid containing ENMs and dissolved molecules, which are then transported to endocytic vesicles (Lim and Gleeson, 2011). Except for brain microvessel endothelial cells, almost all cells show macropinocytosis (Kuhn et al., 2014).

## ENMs and Biological Interactions

### The ENMs-Protein Corona Complex

**Figure 3:**
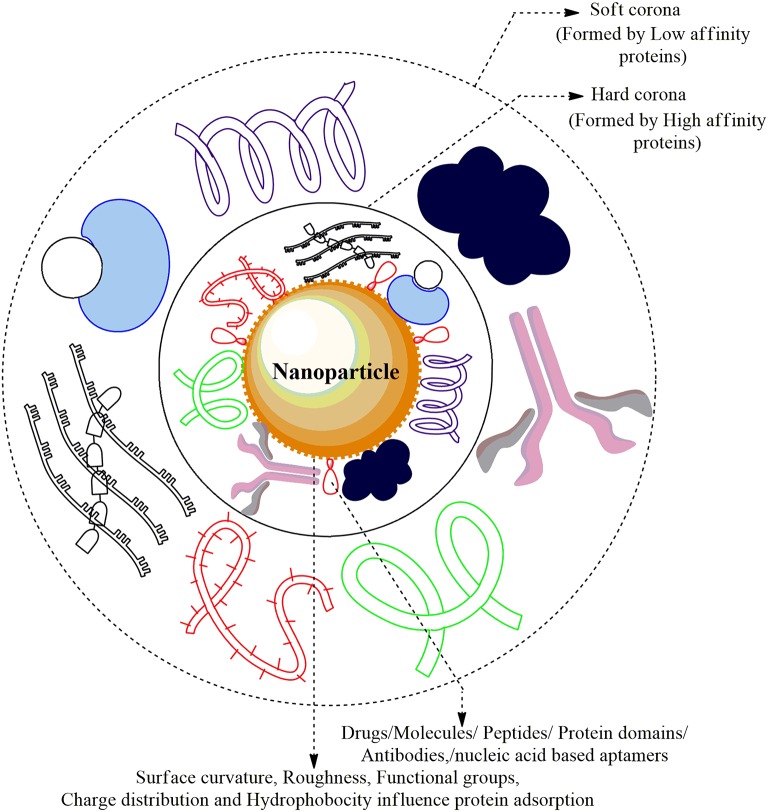
Nanoparticle-Protein Corona (NP-PC) Complex: The cellular proteins in the plasma are adsorbed and form a sheath around the surface of ENMs forming Protein Corona. The proteins that are first to cluster around the NP are higher in abundance/mobility/affinity and form the Hard Corona while the low affinity/mobility proteins form Soft Corona over time. The Plasma proteins forming a coat around the corona are albumin, alpha-2 macroglobulin, immunoglobulin G1, apolipoprotein A-1 drugs, and several small molecules.

After systematic internalization into the biological tissues, ENMs are exposed to various biological fluids and form a dynamic ENM-protein corona complex. This complex exemplifies the “real identity” of ENMs in a biological entity, thus this interaction should be carefully examined to envisage and control the fate of ENMs, including systemic circulation, biodistribution, and bioavailability and clearance (Tenzer et al., 2013; Liu et al., 2015). Upon exposure with the active bio-molecules within the cells/tissues, a “crown” or corona is formed around the ENMs, transforming these ENMs with a biological component i.e., corona (PC) as shown in Figure 3. Although, PC is primarily composed of proteins, and the involvement of other bio-molecules i.e., nucleic acids, sugars, and lipids are not yet documented. The physicochemical properties of ENMs are greatly altered by the adsorption of proteins on ENMs, and ENM-PC complex formation alters the size, surface charge, surface composition, and functional groups of ENMs, thus giving them a new biological identity (Tenzer et al., 2013; Liu et al., 2015). A variety of cellular responses including cellular uptake, fibrillation, circulation time, bioavailability, and even toxicity are determined by the ENM-PC complex, not by the bare ENMs. Therefore, a proper understanding of the interaction between ENM-PC complex and cellular processes is fundamentally important for the identification of a potential model of nanotoxicity. It is quite evident that different corona profiles are produced by different characteristics of ENMs. The ENM-PC complexes can control cellular behavior, bioavailability, and biological responses, and possess many unique physicochemical and biological characteristics. ENMs are excellent carriers for targeted drug delivery because of their specific structural properties, large surface/volume ratio, the capability to append specific agents on their respective surface, the potentiality to cross cellular and tissue barriers, in addition to their long circulation time in the blood. These features significantly contribute to the protein corona formation and is an unavoidable phenomenon. Generally, the protein corona forms around the hydrophobic ENMs because these materials present a greater surface area for protein adsorption and may cause agglomeration and higher opsonization leading to shorter systemic circulation time in blood than the hydrophilic ENMs. The hydrophilic ENMs although, with a lower capacity of protein adsorption, can bind proteins when exposed to the cellular media. The adsorption of proteins on the surface of ENMs and their biological responses are decided by many factors, such as the presence of functional groups and other distinct mechanical properties. In an experiment testing the IgG adsorption behavior and phagocytic efficiency of emulsion droplets and solid polystyrene NPs, it was seen that IgG gets homogeneously distributed around the polystyrene beads independent of its density. In case of its low densities, IgG was concentrated in the interface between the emulsion droplets and the cell, while at higher densities, IgG clusters were seldom visible. This is a clear indication that the emulsion droplets allowed the adsorbed proteins to diffuse and relocate at the interface. Hence, even low quantities of protein can do the job by using the emulsion formulations, while achieving the same effect.

### ENMs and Their Structure-Activity Relationships

Structure-Activity Relationship (SAR) is the correlation between the physicochemical characteristics of ENMs with their biological activities. This allows the prediction and modification of the activity of ENM by manipulation of the molecular structure. The functional or chemical groups responsible for evoking the biological effect can be determined through the analysis of SAR. The SAR approach can also be used to model the potential hazards of ENMs and predict the potential risks specific to ENMs based on specific structural and compositional features (Oksel et al., 2015). The major physicochemical properties that are assessed for their contribution to the bio-effects include length, diameter, thickness, surface reactivity, aspect ratio, zeta potential, biotransformation, hydrodynamic size, surface area, and ability to catalyze ROS generation. A combinatorial Fe_2_O_3_ library with precisely controlled size and shape revealed that cell migration is determined by surface reactivity; inflammatory effects of Fe_2_O_3_ nanorods and nanoplates are controlled by particle properties, metabolite, and protein changes, AR and surface reactivity (Cai et al., 2018). Engineered carbonaceous nanomaterials (ECNs) like SWCNTs, MWCNTs, graphene, and graphene oxides possess high conductivity, tensile strength, surface area, flexibility as well as hydrophilicity and dispersibility in aqueous solutions. It was observed that surface charge, aspect ratio, dispersion state, and surface reactivity are the major contributors of CNT-induced lysosomal damage, cathepsin B release and NLRP3 inflammasome activation in macrophages leading to acute or chronic lung damage (Wang et al., 2017).

## Types of Epigenetic Modifications

**Figure 4:**
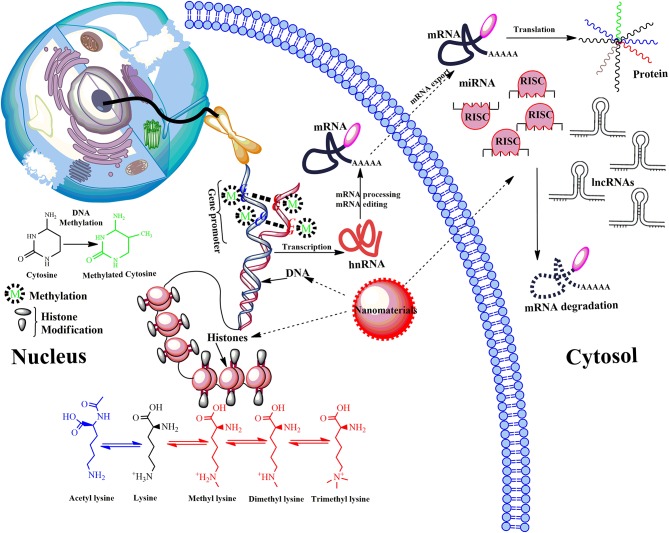
Model showing main epigenetic events that can de-regulate gene expression upon ENMs exposure. Epigenetic mechanisms include post-translational modifications via DNA methylation, the covalent modification (methylation and acetylation) of histone tails and activity of non-coding RNA (ncRNA). DNA methylation down-regulate transcription by blocking the binding of transcription factors to the gene promoter. Modifications of histone tails typically create gene promoters accessible/inaccessible to transcription factors by relaxing/promoting the binding of DNA from around the nucleosomes, therefore up/down-regulating gene expression thus determines the transcriptional profile of neighboring genes. Another mechanism involves microRNAs (miRNA) and long ncRNAs (lncRNAs). miRNAs usually bind via a complementary sequence on a specific target messenger RNA (mRNA), binding induces cleavage or degradation of mRNA or block translation. Many others can bind with chromatin-modifying proteins and recruit their catalytic activity to specific sites in the genome. LncRNAs function in chromatin remodeling, transcriptional regulation, post-transcriptional regulation, they are also precursors for small interfering RNAs (siRNAs). De-regulation of post-translational modifications can result in aberrant gene expression, which causes disease progression.

Epigenetic modifications are stable and heritable alterations mainly driven by three tightly regulated and interconnected processes: (i) DNA methylation, (ii) modification of histones, and (iii) regulation of non-coding RNAs (ncRNAs) that alter DNA accessibility and chromatin structure, thereby modulating the gene expression pattern (Figure 4). These processes are extensively studied and reviewed elsewhere (Strahl and Allis, 2000; Robertson, 2005; Portela and Esteller, 2010; Chervona and Costa, 2012).

In the process of DNA methylation, the covalent linking of a methyl group at the C5 position of cytosine residues in CpG dinucleotide sequences is one of the principal epigenetic tags found in the DNA and it leads to transcriptional silencing of the repeat elements, transposons, and other genes by blocking DNA recognition and binding by certain transcription factors. It has also been observed that factors like methyl CpG binding protein 2 (MeCP2) bind to the methylated DNA to repress the transcription (Hendrich and Bird, 1998) by recruiting histone-modifying enzymes such as histone deacetylases (HDAC), which promotes chromatin condensation (Robertson, 2005). The enzymes involved in the *de novo* methylation and maintenance of the DNA are a family of DNA Methyl Transferases (DNMTs) of which DNMT1 is predominantly responsible for maintaining CpG methylation by adding methyl groups to the non-methylated daughter strand formed during replication, while enzymes like DNMT3a and DNMT3b are required during embryogenesis for methylation (Cirio et al., 2008). The process of demethylation is more complex and can be passive or active. Ten-eleven translocation 2 (TET) family proteins are able to oxidize the methylated DNA into respective carboxycytosine, formylcytosine, and 5-hydroxymethylcytosine (Robertson, 2005).

The four canonical histones; H2A, H2B, H3, and H4 make up the nucleosomes and H1 as a linker, and these histones form the foundation of chromatin. Initially, histones were considered as a static scaffold for DNA packaging but recent findings reveal that they affect the chromatin condensation and DNA accessibility, by tightly regulated post-translational modifications (PTMs). PTMs can guide the state of chromatin (i.e., active vs. inactive) and eventually the expression of genes (Strahl and Allis, 2000). Histones are modified post-translationally by acetylation, methylation, and phosphorylation of tail regions and are extensively studied the phenomenon of PTMs. However, histones may also be modified in other processes, such as citrullination, ubiquitination, ADP-ribosylation, deamination, formylation, O-GlcNAcylation, propionylation, butyrylation, crotonylation, and proline isomerization (Esteller, 2008). Increment in acetylation of histone tails is usually associated with transcriptional activation of genes, while the functional consequences of methylation depend on the number of methyl groups, the residue itself, and its location within the histone tail. The addition or removal of post-translational modifications from histone tails is fairly dynamic and is achieved by a number of different histone modifying enzymes. The enzymes involved in so-called “writing” and “erasing” these reversible marks include, histone acetyltransferases (HATs), histone deacetylases (HDACs), histone methyltransferases (HMTs), histone demethylases (HDMs), histone ubiquitinating enzymes as well as deubiquitinating enzymes, and can either be specific (i.e., histone methyltransferases and demethylases) or general (i.e., HATs and HDACs) in their ability to recognize and alter the amino acid residues of histone tails (Chervona and Costa, 2012).

The third type of epigenetic regulation is mediated by ncRNA, identified relatively recently and is an emerging research field. An ncRNA is a functional RNA molecule that is transcribed from DNA but not translated into proteins. Those ncRNAs that appear to be involved in epigenetic processes can be divided into two main groups; the short ncRNAs (<30 nucleotides) and the long ncRNAs (>200 nucleotides). The three major classes of short non-coding RNAs are microRNAs (miRNAs), short interfering RNAs (siRNAs), and piwi-interacting RNAs (piRNAs). Generally, ncRNAs function to regulate gene expression at the transcriptional and post-transcriptional level by inducing heterochromatin formation, histone modification, DNA methylation targeting, and gene silencing (Ohnishi et al., 2010). siRNAs and miRNAs target gene promoters and direct transcriptional gene silencing by recruiting specific proteins and epigenetic remodeling complexes that suppress gene expression by promoting histone methylation (H3K9 and H3K27), DNA methylation and histone deacetylation (Kim et al., 2006). It has also been reported that long-non coding RNAs play a pivotal role in suppressing transcription by recruiting RNA-binding proteins that interfere with histone deacetylation (Nagano and Fraser, 2009).

## Epigenetic Effects of Engineered Nanomaterials

Nanomaterials can induce epigenetic changes at DNA, RNA and protein levels. The epigenetics of gene expression patterns can be regulated through covalent modification of DNA, histones, small non-coding RNAs (miRNAs, siRNAs, piRNAs) and long non-coding RNAs (lncRNAs). The persistence of these changes through the process of cell division would ensure the alteration of heritable gene expression pattern (Tabish et al., 2017). The normal growth and cellular functions are dependent upon the epigenetic control of gene expression programs. The different epigenetic modulations are shown in Figure 4 are in intercommunication within and between different regulatory mechanisms rather than working independently. The alteration in the DNA methylation of the global or gene-specific sites in the nucleus shows a profound impact on the remodeling of chromatin and their respective locus-specific expression of genes. The testing of epigenetic toxicity potential of ENMs or heavy metals acting as epimutagens has revealed that their exposure can have promising effects on the epigenome yet its contribution in disease development is unclear (Stoccoro et al., 2013). Some of the recent findings have raised agitation regarding the possible epigenetic toxicity and health effects induced by ENMs (Mytych et al., 2017; Smolkova et al., 2017). There are several such *in vivo* and *in vitro* studies which report about the alterations caused by ENMs and studies on histone modifications and expression of miRNAs further enhance our understanding of ENM-induced epigenetic changes. Significant epigenetic effects have been observed based on physical properties i.e., shape and size of ENMs affecting both at the sub-cytotoxic and sub-genotoxic ENM concentrations (Smolkova et al., 2017). The expression of genes and proteins can be grossly affected by these epigenetic changes leading to serious health implications. The epigenetic effects of different ENMs commonly used in disease diagnosis and drug delivery are summarized below.

### ENMs Mediated DNA Modifications

Methylation of DNA (other than histone methylation) is a well-studied epigenetic signaling tool that cells use to lock the genes in the silenced state. Researchers have learned a great deal about DNA methylation, including how and where it occurs. It has also been discovered that methylation of DNA is an important component in numerous cellular processes, including embryonic development, genomic imprinting, inactivation of X-chromosome, and maintenance of chromosome stability. Hypermethylation of promoter regions (CpG islands) silences the DNA repair, cell cycle, and apoptosis pathways genes, whereas hypomethylation of a CpG dinucleotide in the global genome activates gene expressions (Robertson, 2005). Researchers have also observed that errors in DNA methylation/demethylation were associated with a variety of disturbing consequences, including several human diseases such as cancer. DNA hyper- and hypomethylation play a significant role in cancer progression. It was well-established that DNA hypermethylation often silenced the tumor suppressor genes in cancer cells, while the DNA is hypomethylated in cancer cells as compared to normal cells (Robertson, 2005). ENMs that are commonly used in various biomedical applications were shown to induce epigenetic toxicity by promoting alteration in DNA methylation profile. We summarized these *in vitro, in vivo* studies or clinical samples showing DNA methylation in Table 1.

**Table 1:** Summary of the key findings asserted by several *in vitro, in vivo* studies and clinical samples displaying epigenetic changes through DNA modifications induced by the exposure of engineered metallic and non-metallic nanomaterials.

**NPs and functionalization**	**Characteristics size (nm), zeta potential**	**Experimental setup and exposure time**	**Biological model**	**Epigenetic effects**	**Year**	**References**
Gold (Colloidal AuNPs coated with citrate)	5, 60, and 250 nm	*In-vivo* 48 h	BALB/c mice (Single Intratracheal administration)	Lung tissue; hypomethylation of GPX and several genes, hypermethylation in ATM, CDK, and GSR genes	2017	Tabish et al., 2017
Gold (Colloidal AuNPs coated with citrate)	5, 60, and 250 nm	*In-vivo* 48 h	BALB/c mice (Single Intratracheal administration)	Lung tissue; hypomethylation of GPX and several genes, hypermethylation in ATM, CDK, and GSR genes	2017	Tabish et al., 2017
AgNPs using extracts of *Bacillus cereus*	8 nm	*In-vivo* Intravenous infusion at 1 mg/kg doses at 6.5 days postcoitum	8 week mice with ICR (imprinting control region)	In placenta tissue, decreased ZAC1 gene promoter DNA methylation	2015	Zhang et al., 2015
AgNPs	50 ± 5.0 nm (TEM) 55 ± 6.0 nm (water) −25.2 ± 0.1 mV	*In-vitro* 24 h	Mouse Embryonic Fibroblast Cells (NIH3T3)	Apoptosis and nucleosome assembly gene expression alterations Gene ontology analysis revealed alterations in nucleosome assembly and DNA methylation	2018	Gurunathan et al., 2018
ZnO	90 nm	*In-vitro* 48 h	HEK-293 cells	Enhanced expression of TET1 and TET2 genes beside reduction in 5-mC and escalation in 5-hmC content	2017	Choudhury et al., 2017
ZnO	<100 nm	*In-vitro* 24 and 48 h	MRC5 cells	DNA hypomethylation followed by DNMTs activity decline beside reduction in expression levels of endogenous DNMT1 and 3A	2016	Patil et al., 2016
ZnO	<100 nm −12.46 ± 0.28 mV	*In-vitro* 6 and 24 h	Hamster lung fibroblast (V-79) cell lines	HGPRT gene showed a remarkable increase in the mutation frequency along with DNA damage	2019	Jain et al., 2019
CuO	58.7 nm −21.4 ± 1.60 mV	*In-vitro* 24 h	Human small airway epithelial cells (SAEC) and human and murine macrophages (THP-1 and RAW264.7)	L1 and Alu showed hypermethylation, ORF1, ORF2, SINE B1, and SINE B2 reactivated in RAW264.7. Alterations reported in the gene expression of DNMT1 and TET3	2016	Lu et al., 2016a
CuNP	40–60 nm −30.3 mV (PBS) −38.3 mV (pH5)	*In-vivo* 4 weeks through diet	Male albino Wistar rats	A significant decline was observed in the level of global DNA methylation. Reduction in dietary Cu enhances global DNA methylation	2018	Ognik et al., 2019
Anatase TiO_2_	22.1 nm −4.47 ± 0.409 mV	*In-vitro* 24 h	A549 cells	PARP1 promoter hypermethylated	2015	Bai et al., 2015
TiO_2_	21 nm	*In-vitro* 24 h	THP-1, RAW264.7 and SAEC	Methylation levels of SINE 1 and expression of TET2 were enhanced	2016	Lu et al., 2016a
TiO_2_	<100 nm	*In-vitro* 24 and 48 h	MRC5 cells	Hypomethylation of the DNA and reduction in the DNMT activity as well as expression levels of endogenous DNMT1, 3A, and 3B	2016	Patil et al., 2016
TiO_2_	<100 nm	*In-vitro* 24 h	HaCaT cell line	Methionine deficiency and perturbation in the methylation cycle	2013	Tucci et al., 2013
TiO_2_	25 nm (nanotube morphology) or 60 nm (Anastase Morphology)	*In-vitro* 48 h	Human Bronchial Epithelial (16 HBE) and A549 cells	Anatase-type NPs led to a decline in global DNA methylation. Expression levels of methylation related genes and proteins were also altered causing epigenomic toxicity	2017	Ma et al., 2017
SiO_2_	15 nm	*In-vitro* 24 h	HaCaT cell line	DNMT 1, DNMT 3a and MBD2 gene and protein expression showed a dose-dependent decline. Global hypomethylation observed	2010	Gong et al., 2010
			HaCaT cell line	Hypermethylation of PARP-1 and repression of gene expression	2012	Gong et al., 2012
			Primary and immortalized (BEAS 2B) human bronchial epithelial cells exposure over 30 passages	Promoters of 32 genes showed Differentially Methylated Regions. CREB3L1 and BCL2 DNA showed hypermethylation	2014	Zou et al., 2016
Carbon Nanotubes C60 MWCNTs	1 nm	*In-vitro* 24 h	A549 cells	Global DNA methylation levels were significantly elevated.	2016	Li et al., 2016
SWCNTs	1.2–1.5 nm	*In-vivo* 48 h	Male BALB/c mice, single intratracheal administration	The promoter of the ATM gene showed little hypomethylation	2017	Tabish et al., 2017
MWCNTs	5–15 mm long, 27 nm diameter	*In-vivo* Acute (24 h) and Subchronic (7 days) post-exposure	C57BL/6 mice	Increased IFN-γ and TNF-α gene expression (due to hypomethylation of the promoter), decreased Thy-1 (hypermethylation of the promoter) Both lung and blood showed global hypomethylation	2016	Brown et al., 2016
SWCNTs and MWCNTs	SWCNTs (2 nm) MWCNTs (2-100 nm)	*In-vitro* 24 h	THP-1 cells	CNTs induced gene promoter-specific altered methylation leads to hypomethylation of 1,127 different genes	2016	Öner et al., 2016
Nano-Hydroxyapatite	100 × 10 nm	*In-vitro* 72 h	Murine bone marrow stromal cells (BMSCs), Pre-osteoblast MC3T3-E1 cells and Murine osteocyte, MLO-Y4 cells	Pro-osteoblastic marker genes ALP, BSP, and OSC are down-regulated, while upregulation in OPN	2015	Ha et al., 2015
Anionic cadmium telluride QDs (CdTe-QDs)	2.2 nm (green-emitting) and 5.2 nm (Red emitting)	*In-vitro* 24 h	PC12 and N9 murine microglial cells	Cell death characterized by the condensation of chromatin and blebbing of membrane	2005	Lovrić et al., 2005
Modified nano-graphene quantum dots (M-GQDs)	5–15 nm	*In-vivo* 7 days	Zebrafish	Increase in global DNA hypermethylation.	2019	Hu et al., 2019
GQD	3.5 nm	ssDNA	APC gene sequence	Methylated DNA showed B to A structure transition	2019	Rafiei et al., 2019
SWCNTs and MWCNTs		*In-vitro* 24 h	16 HBE cells	SWCNT: DNMT1, NPAT/ATM, PIK3R2 and MYO1C showed prominent changes in sequence-specific methylation in at least one CpG site MWCNT: HDAC4, NPAT/ATM, MAP3K10 and PIK3R2 showed prominent changes in sequence-specific methylation in at least one CpG site	2018	Ghosh et al., 2018
Chiral Au nanoclusters capped with GSH	4–5 nm	*In-vitro* 24 h	Human gastric cancer (MGC- 803) cell line and Human embryonic kidney (HEK 293FT) cell line	TET proteins gene downregulation and decrease of 5-hydroxymethylcytosine and Histone Deacetylase (HDAC) activity	2016	Ma et al., 2016
MWCNTs	(200–100 nm agglomerates)	Clinical samples	MWCNTs exposed workers (*n* = 24) from a factory and unexposed controls (*n* = 43)	Remarkable changes in the methylation of CpG sites in the promoter region of DNMT1, HDAC4, NPAT/ATM, and SKI were observed	2017	Ghosh et al., 2017
SWCNTs and MWCNTs		*In-vitro* 24 h	16 HBE cells	MWCNT: HDAC4, NPAT/ATM, MAP3K10 and PIK3R2 showed prominent changes in sequence-specific methylation in at least one CpG site	2018	Ghosh et al., 2018

In an experiment conducted on HT22 mouse hippocampal neurons, Ag-NPs exposure stimulated the DNA damage response through oxidants alongside changes in methylation patterns of DNA. A significant increment has been reported in the expression levels of DNA methyltransferase (DNMT1, 2, 3a, and 3b) and 5-hmC (Mytych et al., 2017). A significant DNA hypermethylation was reported in Bcl-2 and CREB3L1 and 5-aza (methyltransferase inhibitor), proving that hypermethylation is linked with Bcl-2 and CREB3L1 mRNAs downregulation. The results of this study showed that SiNPs triggered the mitochondrial-mediated apoptosis through PI3K/Akt/CREB/Bcl-2 signaling pathway and its long term exposure can lead to cancer progression (Zou et al., 2016). Oral administration of PVP-coated Ag in mice induces genomic instability and DNA damage in multiple tissues, such as in peripheral blood and/or bone marrow and developing embryos, and which may cause permanent genome alterations leading to cancers (Kovvuru et al., 2015). Nallanthighal et al. orally administrated citrate-coated Ag in wild type and Ogg1 (8-Oxoguanine DNA glycosylase 1) deficient mice showed genotoxicity in both the strains (Nallanthighal et al., 2017). The Ogg1 deficiency showcased exacerbated DNA damage repair. The data suggest that humans with polymorphisms and/or mutations in OGG1 gene are susceptible to Ag ENM mediated damage, which may lead to cancers.

In a study on workers (*n* = 24) with occupational exposure to multi-wall carbon nanotubes (MWCNT) and unexposed controls (*n* = 43) from the same workplace in the blood cells have shown changes in the DNA methylation (Ghosh et al., 2016, 2017). They observed significant methylation changes in DNMT1, ATM, SKI, and HDAC4 promoter CpGs of MWCNT exposed workers, which proves the fact that these occupational exposures may cause epigenetic changes which could produce deleterious effects in future, which may be inherited to the next generation as well. Additionally, these epigenetic misregulations through ENMs may also lead to some lifestyle diseases, which is the critical link for the genotype and phenotype modulations. Although CNTs alone can't bind the DNA, its functionalization (both covalent and non-covalent) with positive charge can effectively condense DNA (Zhou et al., 2013). Hence functionalization of CNTs can lead to epigenetics effects, which need to be studied in detail for further validation in biological systems. Additionally, Amine-modified graphene QDs (AG-QDs) have the ability to enter the nucleus and intercalate with the DNA and it has been known that the intercalating agents have the ability to inhibit the DMNTs, which leads to alterations in genomic DNA methylation patterns leading to epigenetic changes (Lu L. et al., 2016; Castillo-Aguilera et al., 2017; Xu et al., 2018). Hence further experiments are needed in this direction to arrive at the conclusion regarding probable graphene QDs inhibition of DMNTs which may finally affect the cellular environment through epigenetic changes.

BALB/c mice intra-tracheal administration of citrate-coated AuNPs (5, 60, and 250 nm) resulted in both hypomethylation of GPX gene and hypermethylation of ATM, CDK, and GSR genes in the lung tissues (Tabish et al., 2017). The proteins coded by ATM, CDK, and GSR genes regulate the cell cycle, DNA damage sensing, and transcription in response to several intra- and extra-cellular signals whereas GPX gene encodes an enzyme that helps in reducing oxidative stress and retain the redox homeostasis inside the cell (Deponte, 2013; Malumbres, 2014). Hence, hypomethylation and hypermethylation in their respective genes may lead to lung cancer through epigenetic effects. It was reported that DNA demethylation patterns can also be altered by antioxidant-based chiral AuNPs by directly affecting key DNA demethylating enzymes. AuNPs have the ability to alleviate mRNA expression of TET1 and TET2 alongside up- and down-regulation of different miRNAs, a decreased 5-hmC and HDAC activity (Ma et al., 2016). Upon CuO-NPs administration, LINE-1 methylation has been reduced in RAW264.7, while THP-1 and SAEC cell line showed modestly increased methylation profile in Alu and LINE-1 sequences. The reduction in LINE-1 methylation due to CuO-NPs exposure caused enhanced Alu-1 and SINE repetitive elements transcription followed by TET1, TET2, and TET3 expression reduction in mouse macrophages (Lu et al., 2016b). Based on these studies, we can conclude that ENMs trigger de-regulation of genes involved in DNA methylation/demethylation reactions, as well as changes of gene-specific methylation of tumor suppressor genes, inflammatory genes, and DNA repair genes, eventually leading to cancer growth and development. Gold nanorods (GNR) were reported to be nontoxic, and gain access to the cytoplasmic vesicles following endocytosis without any nuclear localization (Chithrani et al., 2006; Qiu et al., 2010). On the contrary, a study by Hauck et al. reported alteration in gene expression by an unknown mechanism following GNR exposure (Hauck et al., 2008). But a recent finding by Ho et al. concluded that the intracellular speciation of GNR alters the dynamic microenvironment by their interactions within the nucleus leading to structural alterations in genomic DNA, which may trigger changes in the gene expression in cells due to the modified oxidation state of Au [Au (0) to Au (I)] (Ho et al., 2018). In another study, Conde et al. showed that the ENM of Au with PEG and protamine (AuNP-PEG-Prot) can modify the plasmid DNA topology acting like a histone-mimetic, affecting the DNA condensation and decondensation in addition to altering DNA conformation and encouraging structural changes (Conde et al., 2012). Nash et al. showed that any alteration in the charge of the AgNPs causes DNA bends through periodic variation in groove widths and depths, while RNA bends through the expansion of the major groove (Nash et al., 2015). All these results sum up the fact that the gold ENMs have a major influence on the microenvironment of nucleus leading to the epigenetic changes.

### ENMs Mediated Histone Modifications

It is well-established that PTMs of histones mediates a variety of essential biological processes, usually via chromatin remodeling leading to expression or repression of target genes (Dong and Weng, 2013). Incorrect targeting of histone-modifying enzymes, such as HDACs, HATs, HMTs, and HDMs, is often accountable for the abnormal PTMs of histone. HDACs, for example, are often found to be over-expressed in different cancers. HDAC1 was shown to be associated with the tumor suppressor retinoblastoma protein (Rb), and in cooperation with Rb lead to the repression of transcription factor E2F-regulated promoter of the gene encoding the cell-cycle protein cyclin E. Abnormal regulation of histone methyltransferases or demethylases in cancer cells also contributes to abnormal histone PTMs patterns (Chervona and Costa, 2012). The effect of ENMs on histone PTMs has been less studied as compared to DNA methylation. However, some researchers suggest that histone modifications are also important molecular targets to understand the toxicity mechanism of different types of ENMs. For example, a study on mouse erythroleukemia cells when exposed to AgNPs, showed a significant reduction in the methylation at lysine residues H3K4 and H3K79 on the b-globin locus, which decreases the histone methyltransferase DOT-1L and MLL levels as well as the direct binding between AgNPs and H3/H4, which finally decreased the hemoglobin production (Qian et al., 2015). The exposure of AgNPs on HaCaT cells, Human lung and breast adenocarcinoma cells showed enhanced phosphorylation of histone H3 at serine 10 (p-H3S10) in a mitosis independent manner due to activation of Aurora Kinase. It has been found that AgNPs induce the formation of globular actin in a dose-dependent manner after incorporating into the inner cells followed by activation of Aurora Kinase (Zhao et al., 2017) and A549 cells showed enhanced phosphorylation at 10th serine residue in H3(p-H3S10) involving MAPK pathway (Zhao et al., 2019). In a study by Gao et al. (2016), enhanced H3K9 methylation and a decrease in H4K5 acetylation were seen on exposure of ZnO to the HaCaT cells. Anionic CdTe-QDs, when exposed to THP-1 cells for 4 and 24 h, exerted their binding to core histones changing their physical and chemical properties leading to an enhanced aggregate formation (Conroy et al., 2008). While, the uncharged CdTe-QDs showed global hypoacetylation in the histone 3 leading to chromatin decondensation in MCF-7 at 4 and 24 h (Choi et al., 2008). When human recombinant histone deacetylase 8 enzyme-treated by a colloid solution of gold, the Au binds -SH group on the surface of enzyme and decreases its activity, which may lead to compromised function in the cells leading to epigenetic changes (Sule et al., 2008). The exposure of Au particles on HeLa cells enhances the connection of core histones and lamin protein due to modulation of heterochromatin (Mazumder and Shivashankar, 2007). Zhang et al. reported that the exposure of SiO_2_ on the A549 cells decreases SIRT6 expression, leading to the upregulation of FST levels due to suppressed deacetylation of H3K9 and H3K56 at FST promoter (Zhang L. et al., 2018). It is concluded from this study FST transcription is negatively regulated by SIRT6 and participates in the regulation of cell particle during SiO_2_ exposure. All these studies shown in Table 2 have clearly observed the histone-based modifications leading to altered promoter expressions with a little or no knowledge on gene enhancers due to their respective ENMs exposures, which are mainly involved in regulating development and cell differentiation.

**Table 2:** Summary of the key findings asserted by several *in vitro* and *in vivo* studies displaying epigenetic changes through histone modifications induced by the exposure of engineered metallic and non-metallic nanomaterials.

**NPs and functionalization**	**Characteristics size (nm), zeta potential**	**Experimental setup and exposure time**	**Biological model**	**Epigenetic effects**	**Year**	**References**
Au particles	5 nm	*In-vitro* 1 h	HeLa cells	Heterochromatin modulation connects core histone and lamin protein	2007	Mazumder and Shivashankar, 2007
A colloid solution of gold	10 nm	Human Recombinant histone deacetylase 8 enzyme		Binds -SH group on the surface of the enzyme and decreases its activity	2008	Sule et al., 2008
Negatively charged (citrate-capped) and positively charged (cysteamine-capped) AuNPs	212.7 nm−38.7 mV	*In-vitro*	Triple-negative breast cancer (MDA-MB-231 and MDA-MB-468) cells	–ve charged NFPs; increased the expression of MKP-1, dephosphorylated and deacetylated histone H3 at Ser10 and K9/ K14 residues respectively +ve charged NPs; decreased the expression of MKP-1, phosphorylated and acetylated histone H3 at Ser 10 and K9/K14 residues respectively	2018	Surapaneni et al., 2018
AgNPs with polyvinylpyrrolidone coating	25 nm	*In-vitro* 72 h	Mouse erythroleukemia cells	Methylation of H3 at lysine (Lys) 4 (H3K4) and Lys 79 (H3K79) on the b-globin locus was reduced greatly Decreased, disruptor of telomeric silencing 1-like and mixed lineage leukemia histone methyltransferase levels beside direct binding of AgNPs to H3/H4	2015	Qian et al., 2015
AgNPs	200 nm	*In-vitro* 24 h	Human skin keratinocytes (HaCaT), Human lung and breast adenocarcinoma cells (A549 and MCF-7)	Activation of Aurora kinase, leading to the induction of phosphorylation of histone H3 at serine 10 (p-H3S10) in a mitosis independent manner	2017	Zhao et al., 2017
AgNPs	100 nm	*In-vitro* 10 h	A549 cells	Phosphorylation of histone H3 at serine 10 (p-H3S10) Involves MAPK pathways and independent of DNA damage	2019	Zhao et al., 2019
ZnO	<100 nm	*In-vitro* 24 h	HaCaT cells	H3K9 showed a marked increase in methylation status while H4K5 showed a decline in acetylation. Along with the chromatin condensation, HMT G9a showed up-regulation while HATs GCN5, P300 and CBP were downregulated	2016	Gao et al., 2016
SiO_2_		*In-vitro* 24 h	A549 cells	Decreased SIRT6 expression, leads to the upregulation of FST level due to suppressed deacetylation of H3K9 and H3K56 at FST promoter	2018	Zhang L. et al., 2018
Nanofibrous scaffolds		*In-vitro*	Fibroblasts isolated from ear tissue of C57BL/6 mice	The decrease in HDAC activity, upregulation in the expression of WD repeat domain 5 (WDR5) with increasing H3 methylation and acetylation	2013	Ha et al., 2015
Soft NMs Cholesterylbutyrate solid lipid NPs releasing butyric acid	100–150 nm	*In-vitro and In-vivo*	Cancer cell lines and Rat intracerebral glioma model	Inhibition of HDACs	2008	Brioschi et al., 2008
Soft NMs K- 182 HDACI-coated cationic NPs	137.9–176.7 nm 64.0–63.0 mV	*In-vitro* 24 h	Human prostate cancer (PC-3) cells and human breast cancer (Sk-Br-3) cells	Remarkably high gene expression and hyperacetylation of the core histones	2009	Ishii et al., 2009
Anionic CdTe-QDs	3.4 nm	*In-vitro* 4 or 24 h	THP-1 cells	NPs binding to core histones changes their physical and chemical properties leading to an increase in aggregate formation	2008	Conroy et al., 2008
CdTe-QDs		*In-vitro* 4 or 24 h	MCF-7 cells	Deacetylation of Histone 3 leads to chromatin decondensation (global hypoacetylation)	2008	Choi et al., 2008

### ENMs Mediated De-regulation of Non-protein-coding RNAs

The epigenetic machinery is greatly influenced by the presence of regulatory non-protein-coding RNAs (ncRNAs), which play a crucial part in the regulation of gene expression through their dynamic interactions with DNA, RNA, and proteins leading to various epigenetic modifications (Frías-Lasserre and Villagra, 2017). The regulatory non-protein-coding RNAs (ncRNAs) can be categorized into long ncRNAs (larger transcripts) and short ncRNAs (<200 nucleotides). The different subtypes of short ncRNAs include miRNAs, endogenous siRNAs, and piRNAs. The lncRNAs are natural antisense transcripts, or sense intronic RNAs, or long intergenic noncoding RNAs (Peschansky and Wahlestedt, 2014). These RNAs have a multitude of functions in cells, and it is well-researched that about one-quarter of the human genes engaged are regulated by miRNAs are involved in tumorigenesis, cardiovascular and developmental disorders, neurological and other diseases (Portela and Esteller, 2010; Paul et al., 2018). Non-protein coding RNAs can modify gene expression by interacting with other epigenetic machinery, similar to alteration in DNA methylation by regulation of DNMTs, modulating the expression and function of histone modifier proteins, and/or chromatin remodeling. ncRNAs are highly flexible and dynamic in interacting with DNA, RNA, and proteins (Portela and Esteller, 2010; Paul et al., 2018). These properties make ncRNAs capable of mediating a plethora of epigenetic mechanisms by which the cellular environment will be altered based on various external or internal stimuli. Therefore, it is quite clear that, together with DNA methylation and histone post-translation alteration machinery, regulation of ncRNAs can also be influenced by exposure to ENMs.

Early studies by Halappanavar et al. have established the fact that ENM exposure alters the expression of respective ncRNAs (Halappanavar et al., 2011). Significant changes were observed by them in the expression pattern of 16 miRNAs in lungs tissue of a mouse model subjected to surface-coated nanoTiO_2_. Among another miRNA, the best results were shown by mmu-miR-449 in comparison with the native controls. This research also showed that nanoTiO_2_ induces lung inflammation but failed to create a direct connection between miRNA deregulation and pulmonary injury. In another study by Ng et al. using gold nanoparticles, it was shown that upregulation of miR-155 is naturally accompanied by downregulation of the PROS1 gene coding S protein, a cofactor with great influence on blood clotting processes (Ng et al., 2011). This study gives an insight into the molecular mechanisms and their impact on epigenetic processes through gold nanoparticles toxicity. Significant shifts in miRNA expression and transplacental size-dependent clastogenic and epigenetic effects have been observed through AuNP as reported by Balansky et al. in the mouse fetus (Balansky et al., 2013). They observed upregulated expression of 28 miRNAs in the fetal lung and 8 miRNAs in the fetal liver, with miR-183 and Let-7a upregulated in both the tissues. The cells exposed to Silver NPs showed a negative correlation between the expression of hsamiR-219-5p and TRIB3 and MT1F genes, which are involved in the cell cycle, oxidative stress, and apoptosis (Eom et al., 2014). On the contrary, AuNPs induces MC3T3-E1 bone cell mineralization by altering the expression of targets genes allied with the formation of bone through some specific miRNAs (Mahmood et al., 2011). The exposure of TiO_2_NPs altered the autophagy pathway through long-lasting (48 h) alleviation of miRNA-21 and miRNA-30a expression. It was also observed that after every 2 h miR-155 is upregulated, but is subsequently degraded following longer times of exposure (Alinovi et al., 2017). A day-long exposure of iron oxide NPs (Fe_2_O_3_NPs) in NIH/3T3 fibroblast cells induces a change in the 167 miRNAs expression. These epigenetic, genomic and non-genomic cellular changes which have been observed through ENM exposure are summarized in Table 3. All the *in vivo* and *in vitro* studies have only summed up the fact that ENMs induce alterations in miRNA expression without going deeply into their associated underlying molecular mechanisms and their assisted repercussions. The role of lncRNA, siRNA, and piRNA in the epigenetic modifications during ENM exposure remain largely unknown.

**Table 3:** Summary of the key findings asserted by several *in vitro* and *in vivo* studies displaying epigenetic changes through miRNA alterations induced by the exposure of engineered metallic and non-metallic nanomaterials.

**NPs and functionalization**	**Characteristics size (nm), zeta potential**	**Experimental setup and exposure time**	**Biological model**	**Epigenetic effects**	**Year**	**References**
AuNPs coated with citrate	20 nm	*In-vivo* 1 week and 2 months	Male wistar rats single tail vein injection of 0.2 mL (15.1 mg/mL)	21 miRNAs dysregulation (miR-298 upregulated)	2012	Chew et al., 2012
Colloidal AuNPs coated with citrate	20 nm	*In-vitro* 48 or 72 h	Lung fibroblast (MRC5) cell line	Chromatin condensation, miR-155 upregulation, PROS1 gene downregulation	2011	Ng et al., 2011
Colloidal AuNPs coated with citrate	20 nm	*In-vivo* Transplacental treatment on gestation days; 10th, 12th, 14th, and 17th	Adult female and male Swiss albino mice	Fetus lung: 28 miRNAs dysregulation, let-7, and miR-183 upregulation Fetus liver: 5 miRNAs dysregulation, let-7, and miR-183 upregulation	2013	Balansky et al., 2013
AgNPs	<100 nm	*In-vitro* 24 h	Human Jurkat T cell and Jurkat clone E6-1	63 miRNAs expression altered and MT1F and TRIB3 genes expression is –vely correlated with miR-219-5p	2014	Eom et al., 2014
AgNPs	23 nm	*In-vitro* 24 h	Mouse osteoblastic cells (MC3T3-E1 bone cells)	Altered expression of miRNA resulting in specific gene expression allied with bone formation	2011	Mahmood et al., 2011
Superparamagnetic iron-oxide nanoparticles (SPIONs)	4–7 nm	*In-vitro* 24 h	Rat pheochromocytoma (PC12) cell line	Wide changes in miRNA profile	2015	Sun et al., 2015
Fe_2_O_3_		*In-vitro* 12 and 24 h	NIH3T3 cells	Genome-wide changes in the miRNAs expression profile	2011	Li et al., 2011a
SPIONs	20 nm	*In-vitro* 24 h	human liver carcinoma (HepG2) cells	Altered miRNAs expression but don't affect DNA methylation	2019	Brzóska et al., 2019
Co_3_O_4_ NPs	17 nm −19.1 mV	*In-vitro* 24 h	A549 cells	A lower and temporary downfall in the expression of miR-21, miR-30a. Levels of miR-21 recovered after 24 h while miR-30a showed upregulation. miR-155 levels are high after 2–4 h but decreased on longer exposure	2017	Alinovi et al., 2017
TiO_2_	<100 nm	*In-vitro* 1 h daily for 11 consecutive days	C57BL/6 female mice	Upregulation in the targeting genes involved in immune response in the lungs like miR-1, miR-449a, and miR-135b	2011	Halappanavar et al., 2011
TiO_2_	38 nm	*In-vitro* 24 h	A549 cells	miR-21 and miR-30a showed significant down-regulation along with alteration in miR-155 expression	2017	Alinovi et al., 2017
SiO_2_	70 nm	4, 8, 24, or 72 h after treatment	BALB/c mice	miR-122 and miR-192 showed upregulation induced by nSP70-C	2013	Nagano et al., 2013
MWCNTs		*Invitro* 12 and 24 h	NIH3T3 cells	Wide dysregulation was seen in the expression of miRNAs; three KE GG pathways are remarkably regulated	2011	Li et al., 2011a
CdTe-QDs	1–2.5 nm	*In-vitro* 12 and 24 h	NIH3T3 cells	Global alteration of the expression pattern of miRNAs in cells with apoptosis-like cell death	2011 2013	Li et al., 2011a,b; Sun et al., 2013

## Future Prospects

The ENMs are extensively being utilized in various commercial applications and are a constituent of numerous consumer products. Despite being a commercial commodity, many concerns have been raised regarding the potentially harmful effects of ENMs on the environment and human health. ENMs used in personalized medicines remain associated with several health issues and pose a great concern among researchers worldwide to overcome their toxicity. After entering into the human body, ENMs accumulate in the tissues/organs because of their insoluble, non-biodegradable, non-biocompatible nature, thus restricting the pace of excretion, resulting in long-term exposure and subsequent toxicity. ENMs have a tendency to aggregate and form large structures ranging from 5 to 500 nm in diameter and translocate the lung-blood barrier through an endocytic uptake mechanism and enter the blood circulation. These are transported through the body to various tissues and bodies such as the heart, body, spleen, gastrointestinal tract, cardiac system, and central nervous system where they have damaging impacts on health. Nanomaterials communicate with biomolecules i.e., DNA, proteins, and lipids within the body and altering the fundamental cellular and metabolic processes like cell division, cellular signaling, apoptosis and trigger inflammation epigenetic alteration and modifications in the chromosome. Among various toxicity mechanisms, epigenetic mechanisms are not very well-studied. Here we summarize the toxic effects of various ENMs leading to the epigenetic changes. It was well-established that epigenetic processes shape our development and enable us to adapt to a constantly changing environment. The epigenetic manipulation is associated with several reported disease conditions, such as allergic contact dermatitis, inflammatory bowel disease, childhood asthma, chronic obstructive pulmonary disease, and several type of cancers, neurodegenerative, and genetic disorders. Epigenetic mechanisms are also related with the progression of autoimmune diseases and several other inflammatory disorders like multiple sclerosis, rheumatoid systemic lupus erythematosus, arthritis, psoriasis, Crohn's disease (Smolkova et al., 2018). ENMs are considered potential epimutagens, as they promote neoplastic changes by disrupting epigenetically preserved gene function via global epigenetic processes. Therefore, it is of utmost importance to understand the epigenetic changes induced by ENMs used in personalized nanomedicines. ENM associated toxicity remains a concern in developing the strategies to minimize the negative impacts, thus it is critical to understand why ENMs pose toxicity. Toxicity of ENM is largely dependent on its long-term accumulation in the biological tissues and affinity with biomolecules, which is mainly influenced by functional groups, purity, and size of ENMs. Research activities should be focused on optimizing physicochemical properties from synthesis to purification. Improved *in vivo, in vitro*, and epidemiological study considering modern molecular—omics strategies utilizing modern techniques and platforms would definitely help to identify detailed epigenetic mechanisms that link, or act as molecular markers of, environmental exposures and human health concerns. We anticipate that having a centralized database on ENMs toxicity in the context of physicochemical properties and a well-established *in vivo* system will be an added benefit in understanding the nature and extent of toxicity. Finally, there is an urgent need to develop guidelines on minimum concentrations, which could be possible only by establishing close collaboration between scientists from academia and industries, which should be closely monitored by the governmental and regulatory bodies, promoting the research within a regulatory context. These collaborative activities drive the development and implementation of epigenetically relevant integrated testing strategies and policies for the continued protection of public health.

## Conclusion

Epigenetic alterations have the potential to induce prolonged changes in the programming of gene expression and any failure in the detection of these changes could lead to unexpected and unpleasant effects in the biological system. ENMs have a little yet strong effect on epigenetics, so it becomes mandatory to scrutinize them before their extensive applications specifically in the biomedical field. Further studies on this subject matter seem necessary in order to outline/strategize an effective nano-focused risk evaluation strategy/approach that consists of more substantial information about ENMs and its interaction with the surroundings including human and environment.

## Author Contributions

PB conceived the idea. PB and MG worked on study conception and design. MG, PB, KZ, and PM screened titles for relevance and abstracted the data from the eligible full-text articles. MG, PB, KZ, and PM analyzed and interpreted the data and drafted the manuscript. PB and MG critically revised the manuscript with input from the entire team. All authors have read and approved the final draft.

### Conflict of Interest

The authors declare that the research was conducted in the absence of any commercial or financial relationships that could be construed as a potential conflict of interest.
